# Resequencing Reveals Different Domestication Rate for *BADH1* and *BADH2* in Rice (*Oryza sativa*)

**DOI:** 10.1371/journal.pone.0134801

**Published:** 2015-08-10

**Authors:** Qiang He, Jie Yu, Tae-Sung Kim, Yoo-Hyun Cho, Young-Sang Lee, Yong-Jin Park

**Affiliations:** 1 Department of Plant Resources, College of Industrial Science, Kongju National University, Yesan, Republic of Korea; 2 Seedpia, 85 Maesil-ro, Kwonsun-ku, Suwon, Republic of Korea; 3 Department of Medical Biotechnology, Soonchunhyang University, Asan, Republic of Korea; 4 Legume Bio-Resource Center of Green Manure (LBRCGM), Kongju National University, Yesan, Republic of Korea; Louisiana State University Agricultural Center, UNITED STATES

## Abstract

*BADH1* and *BADH2* are two homologous genes, encoding betaine aldehyde dehydrogenase in rice. In the present study, we scanned *BADHs* sequences of 295 rice cultivars, and 10 wild rice accessions to determine the polymorphisms, gene functions and domestication of these two genes. A total of 16 alleles for *BADH1* and 10 alleles for *BADH2* were detected in transcribed region of cultivars and wild species. Association study showed that *BADH1* has significant correlation with salt tolerance in rice during germination stage, the SNP (T/A) in exon 4 is highly correlated with salt tolerance index (STI) (P<10^−4^). While, *BADH2* was only responsible for rice fragrance, of which two *BADH2* alleles (8 bp deletion in exon 7 and C/T SNP in exon 13) explain 97% of aroma variation in our germplasm. Theses indicate that there are no overlapping functions between the two homologous genes. In addition, a large LD block was detected in *BADH2* region, however, there was no large LD blocks in a 4-Mb region of *BADH1*. We found that *BADH2* region only showed significant bias in Tajima’s D value from the balance. Extended haplotype homozygosity study revealed fragrant accessions had a large LD block that extended around the mutation site (8 bp deletion in exon 7) of *BADH2*, while both of the *BADH1* alleles (T/A in exon 4) did not show large extended LD block. All these results suggested that *BADH2* was domesticated during rice evolution, while *BADH1* was not selected by human beings.

## Introduction

Betaine aldehyde dehydrogenase (BADH) is an enzyme found in a large number of plant species, and its catalyst glycine betaine (GB) is a powerful osmoprotectant associated with salt and drought stress tolerance [1]. In many plant species, such as mangrove, spinach (*Spinacia oleracea* L.), amaranth (*Aramanthus* spp.), barley (*Hordeum vulgare* L.) and sorghum (*Sorghum bicolor* L.), BADH plays a role in abiotic stress tolerance through the accumulation of GB from betaine aldehyde (BA). Conversely, plant species such as tobacco (*Nicotiana tabacum* L.), tomato (*Solanum lycopersicum* L.) and rice (*Oryza sativa* L.) reportedly do not accumulate GB due to insufficient BA [1–3].

Rice is regarded as a typical non-glycine betaine-accumulating species with two functional genes coding BADH (EC 1.2.1.8). *BADH1* (Os04g0464200) is located on chromosome 4 and *BADH2* (Os08g0424500) is found on chromosome 8 [1,4]. Both genes have 15 exons and show 75% sequence homology at the amino acid level [5]. *In vitro* studies showed that rice BADH1 has very low activity on BA but the function and mechanism of *BADH1* are uncertain [6]. The rice *BADH1* has been reported close correlation with salt tolerance [7], while as Singh et al. (2010) argued *BADH1* is associated with rice fragrance [1]. In contrast, *BADH2* has been identified as a recessive gene conferring fragrance in rice, rather than abiotic stress tolerance[8]. The loss of function of *BADH2* is the reason for aromatic rice, accumulating the main flavoring components 2-AP (2-acetyl-1-pyrroline).

Fitzgerald et al. (2008) reported that as the salt concentration increased, the transcript level of *BADH1* increased significantly in leaf tissues of both non-fragrant and fragrant rice, while no consistent relationship was observed between *BADH2* transcript levels and salt concentration during the seedling stage [5]. However, Fitzgerald et al. (2010) found a highly significant difference in the ability to produce mature seed between the fragrant and non-fragrant rice in the presence of salt [2]. Thus, *BADH1* and *BADH2* may be responsible for salt stress tolerance, but at different growth stages.

*BADH2* has been extensively studied at the genetic and molecular levels for rice fragrance. Many studies on the *BADH2* have been reported since Bradbury et al. (2005) found a strong allele with eight base-pairs deletion in exon 7, causing 2AP-dependent strong fragrance in rice [8]. Numerous *BADH2* alleles, at least 15 alleles in transcribed region, have been detected in diverse rice germplasms [8–16]. However, *BADH1* has not been studied as extensively as *BADH2*. Only three single-nucleotide polymorphisms (SNPs) have been reported so far [1].

Investigating the domestication of the agriculturally important gene is one of the methods to enlighten the rice evolutionary history, which are receiving increasing research attention in recent years [11,17–19]. In rice, a number of genes have been proved as the domesticated genes, such as *sh4* [20], *rc* [21], *prog1* [22], *GS3* [23] including *badh2* [11].The *badh2*.*1* allele has been significantly selected during the aromatic rice evolution [11]. The evolutionary path of *BADH1*, however, has not been fully explored. It is of great interest to explore whether rice *BADH1* gene was also involved in the domestication.

The goal of this study were to: (1) identify sequence polymorphism in the *BADH1* and *BADH2* gene from 295 cultivated rice and 10 wild rice by re-sequencing and (2) investigate the function of *BADHs* on the salt tolerance at germination stage and rice fragrance, and their overlapping functions. Lastly, we (3) investigated evolutionary path of *BADHs* in Asian rice.

## Materials and Methods

### Plant materials

Two hundred and ninety-five rice varieties used for the present study were collected from RDA gene bank of Korea. Among the 295 accessions, 137 accessions were selected from worldwide 4,046 accessions, based on 15 SSRs by a heuristic approach using PowerCore software [24,25], the rest 158 accessions were selected from Korean breeding varieties. All rice accessions were planted in a paddy field at Kongju National University, Korea, in 2012 and 2013.

### Salt stress evaluation

Seeds of each accession were sterilized in sodium hypochlorite (1%) solution for 10 min and then washed twice with deionized distilled water. Then, the seeds were placed in petri dishes with two-layer filter paper and soaked in 200 mM NaCl solution, which was previously determined as an the optimal concentration (data not shown) to distinguish salt stress tolerance and sensitivity. The salt tolerance index (STI) was used to evaluate salt stress tolerance, where STI (%) = (total dry weight of the samples/total dry weight of the control) × 100.

### Assay of rice fragrance

The aroma of rice grains and leaves were measured by a sensory evaluation according to the method of Amarawathi et al. (2008) with minor modifications. Ten milled rice grains were placed in a 15 ml tube containing 8 ml of 1.7% KOH and incubated at 30°C water bath for 10 min with lids on. The lids were then opened and samples were smelled. The aroma of young leaves was checked by the same method to confirm the results for rice grain.

### Resequencing of 295 rice accessions and allele detection for *BADH1* and *BADH2*

HiSeq 2000 and HiSeq 2500 were employed for whole-genome resequencing of the 295 rice accessions. Raw sequences were first processed to obtain an average of quality score (QS) per read ≤ 20 by trimming 3'-end of reads using SICKLE (https://github.com/najoshi/sickle). High-quality reads were aligned to the rice reference genome IRGSP-1.0 (http://rapdb.dna.affrc.go.jp/download/irgsp1.html) using the Burrows-Wheeler Aligner (BWA) (version 0.7.5a) with default parameters [26]. The reads that does not meet BWA quality criteria or not match the reference genome were removed. PCR duplicate reads were removed by using PICARD (version 1.88) (http://broadinstitute.github.io/picard/). By using Genome Analysis Toolkit (GATK) (version 2.3.9 Lite) [27], regional realignment and quality score recalibration were carried out. SNPs and InDels were, then, identified with ≥ 3X of read depth coverage. Overall, the mapping depth about 9X in average. Sequence data for 10 WT rice accessions (5 *Oryza rufipogon* and 5 *Oryza nivara*) were downloaded from the National Centre for Biotechnology Information Sequence Read Archive 023116 [19]. The polymorphisms of *BADH1* and *BADH2* were purified from whole-genome resequencing data.

### Association study

Genotypic and phenotypic data files were prepared and imported to TASSEL4.0 [28] for association test. The general linear model (GLM), containing the SNP tested as a fixed effect, was applied to test the association between phenotypic variation (aroma and STI) and 19 alleles, which located in transcribed region of *BADHs* without any missing data. To get reliable results, P-value ≤ 0.001 was considered to have a significant effect on each trait.

### Linkage disequibrium (LD) block detection, nucleotide diversity, Tajima’s D analysis and extended haplotype homozygosity (EHH) analysis

To identify LD blocks in both wild and cultivated rice populations, we used Haploview software with “-dprime–minMAF 0.05 –hwcutoff 0.001 –blockoutput GAB –pairwiseTagging” parameters [29]. Average pairwise divergence within the population was estimated in a 4-Mb interval of *BADH* genes. Sliding windows of 1 kb and 3k were used to estimate nucleotide diversity for the 4-Mb region of each gene. In each window, the π and Tajima’s D value was calculated using VCFtools [30]. The pattern of the results were plotted using R scripts. Extended haplotype homozygosity (EHH) was calculated using the R package rehh [31].

## Results

### Salt stress tolerance and rice fragrance

A total of 205 rice accessions, without any missing sequence information of *BADH1* and *BADH2*, were evaluated for salt stress index and rice fragrance. The average STI is 1.14. Among these, RWG-140 had the highest stress tolerance (STI, 1.50). However, RWG-222 had the lowest STI (0.26), which is salt stress sensitive. In the rice aroma test, total 12 accessions were evaluated to be fragrant rice (S3 Table).

### Polymorphisms of *BADH1* and *BADH2*

In this study, we used the whole genome resequencing data to study *BADH1* and *BADH2*. From our resequencing data set (cultivated rice), seven SNPs were found in the exon region, three SNPs and one insertion were in the untranslated region (UTR) in *BADH1* (S1 Table). For *BADH2*, three SNPs, two deletions and one insertion were located in exon part, while four SNPs and three insertions were detected in untranslated region (S2 Table). When we checked those regions in the wild rice, we found, to our surprise, very high sequence diversity in *BADH1* gene region, including four SNPs in UTR and twelve SNPs in exon region. However, only four heterozygous SNPs were found in *BADH2* exon part and five heterozygous SNPs and one insertion in UTR (S1 and S2 Tables).

Among the 16 exonic SNPs of *BADH1*, only seven were non-synonymous mutations, others were synonymous mutations. In our rice germplasm, five non-synonymous mutations were detected. Here we are using “P1” and “P2” to represent the gene positions in *BADH1* and *BADH2*, respectively. The five mutations are described as follows: 1) one G/A SNP (P1_141_) in exon 1 leading to substitution from arginine to histidine; 2) one T/A SNP (P1_1483_) in exon 4 leading to asparagine to lysine substitution; 3) one C/A SNP (P1_3605_) in exon 11 leading to substitution from glutamine to lysine; 4) one T/C SNP (P1_3612_) in exon 12 leading to substitution from isoleucine to threonine; 5) one G/T SNP (P1_3883_) in exon 12 leading to substitution from glutamic acid to aspartic acid. Two non-synonymous mutations were represented in wild type as heterozygous SNPs. One is T/A SNP (P1_1232_) in exon 3 leading to a substitution from aspartic acid to glutamic acid, the other was a G/T SNP (P1_3672_) leading to a substitution from arginine to leucine in exon 11 (S1 Table).

Eighteen alleles were detected in *BADH2* transcribed region. Ten of 18 were located in coding region, and eight were located in untranslated region. In our materials, 1) 8-bp deletion in exon 7 (P2_3036_) leading to premature transcription termination; 2) one C/A SNP (P2_4488_) in exon 10 leading to amino acid substitution from alanine to glutamic acid; 3) three base-pair deletion (P2_5240_) in exon 12 leading to loss of one amino acid; 4) one C/T SNP (P2_5390_) in exon 13 leading to substitution from alanine to valine; and 5) one base pair insertion in exon 14 resulting in frameshift and premature transcription termination have been detected. One synonymous SNP (P2_4528_) in exon 10 has been found in two varieties. Two non-synonymous SNP were detected in wild rice, 1) C/G SNP (P2_205_) in exon 1 resulting to alanine to glycine substitution; and 2) C/T SNP (P2_2685_) in exon 5 resulting to alanine to valine substitution. Those two alleles were heterozygous and initially detected in *BADH2*. In the UTR, three InDels, P2_14_ (1 bp insertion), P2_36_ (1 bp deletion) and P2_41_ (insertion in the 5’UTR) and two SNPs (P2_24_ C/T in the 3’UTR and P2_6038_ in the 3’UTR) were detected.

### Association analysis of salt tolerance, aroma and *BADHs* alleles

Due to the limitations of whole-genome resequencing, missing sequence data were inevitable. In this study, we sequenced total 295 rice samples, most had high sequencing coverage both in *BADH1* and *BADH2* region, but some accessions had low coverage or missing sequence in these gene regions. After removing accessions with missing sequence data, a total of 205 accessions remained. Among these 205 accessions, totally 19 SNPs and InDels were detected in *BADH1* and *BADH2* transcribed regions (S3 Table), and subsequently used for the association studies (Table 1).

**Table 1:** The association study of salt tolerance index and candidate regions.

Marker	Locus	Locus_pos	Marker_F	Marker_P	Marker_R^2^
rs-01	*BADH1*	46	0.24703	0.7814	0.00295
rs-02	*BADH1*	87	16.44522	7.65E-05	0.08916
rs-03	*BADH1*	101	7.53E-04	0.97814	4.48E-06
rs-04	*BADH1*	181	0.24703	0.7814	0.00295
rs-05	*BADH1*	1483	11.39543	2.30E-05	0.12008
rs-06	*BADH1*	3605	11.39543	2.30E-05	0.12008
rs-07	*BADH1*	3883	0.22252	0.63774	0.00132
rs-08	*BADH1*	4811	11.39543	2.30E-05	0.12008
rs-09	*BADH2*	14	0.05781	0.81028	3.44E-04
rs-10	*BADH2*	24	0.05781	0.81028	3.44E-04
rs-11	*BADH2*	36	6.5564	0.01133	0.03756
rs-12	*BADH2*	41	0.05781	0.81028	3.44E-04
rs-13	*BADH2*	42	0.49606	0.48221	0.00294
rs-14	*BADH2*	49	3.97914	0.0205	0.04549
rs-15	*BADH2*	115	0.49606	0.48221	0.00294
rs-16	*BADH2*	3036	1.22893	0.2692	0.00726
rs-17	*BADH2*	4488	0.05781	0.81028	3.44E-04
rs-18	*BADH2*	4528	0.06933	0.79264	4.13E-04
rs-19	*BADH2*	5390	0.37313	0.54213	0.00222

Locus_pos: Physical distance from the transcription initiation site of the gene.

**Fig 1:**
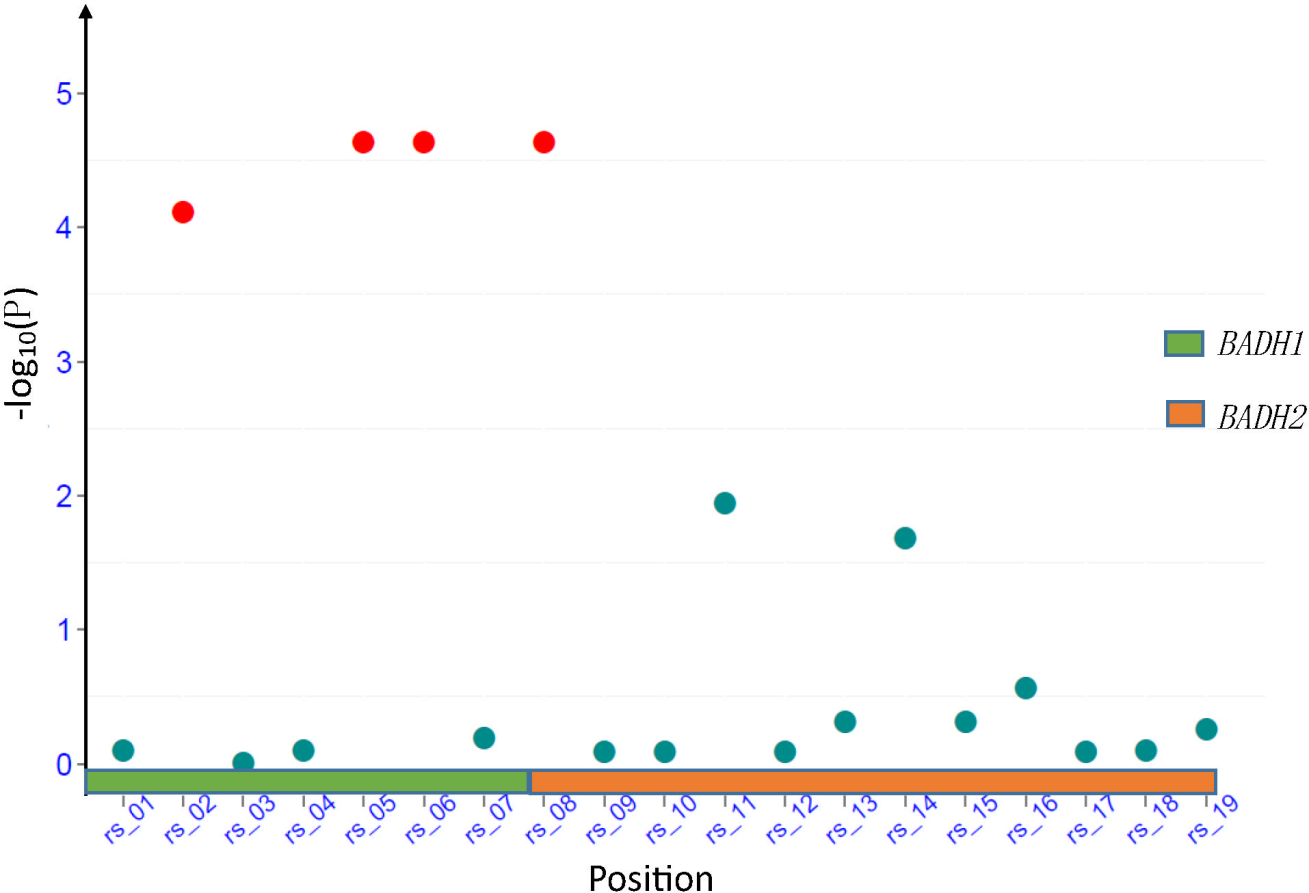
Associations across transcribed regions of *BADH1* and *BADH2*.

The association study demonstrated a relatively high correlation (*R* ^*2*^) of 0.12 between P1_1483_/P1_3605_/P1_4811_ and STI (P<10^−4^) in *BADH1* (Table 1 and Fig 1), while no correlation was observed in *BADH2* in this regard. On the other hand, it was found that two *BADH2* alleles (P2_3036_, P2_5390_) explain 97% of aroma variation in our germplasm. However, no association existed between aroma and *BADH1* (S4 Table). It is suggested that *BADH1* and *BADH2* have different functions in rice.

### Extended haplotype homozygosity study

**Fig 2:**
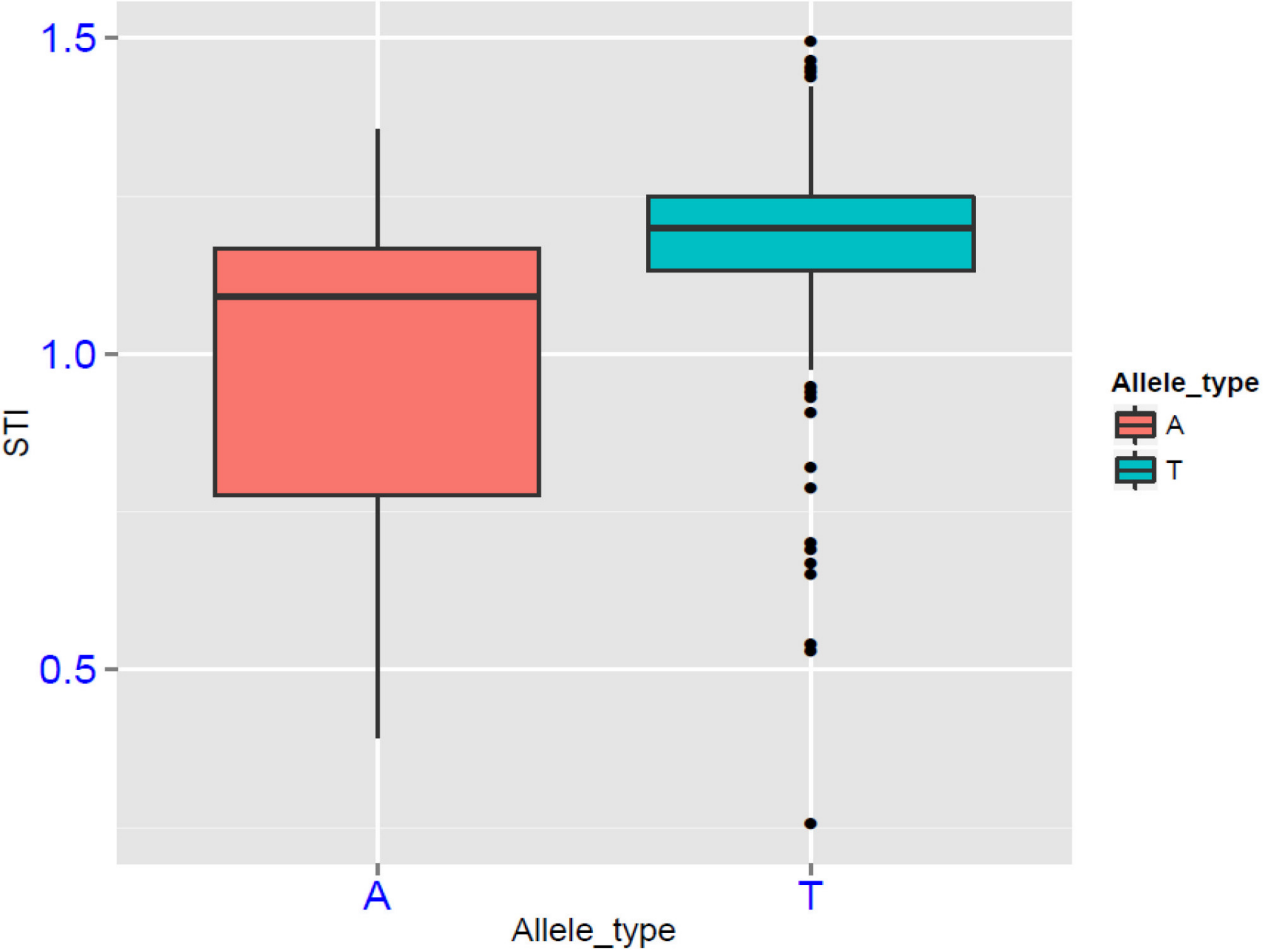
EHH plot across the *BADH1* and *BADH2* genomic region. (a) EHH of *BADH1* genomic region. Wild type (T) and P11483 (A) did not show large LD block. (b) EHH of *BADH2* genomic region. The fragrant accessions carrying the P2_3036_ (*badh2*.*1* which has 8 bp deletion in exon 7) allele exhibited a large block of extended LD around the FNP, while wild type allele declined rapidly.

According to the association study, P2_3036_ (8-bp deletion in exon 7) is the major allele which is associated with rice fragrance (S4 Table). P2_3036_ also called *badh2*.*1* by Kovach et al. (2009). Here we follow the method which was used by Kovach et al. (2009) for domestication study. Firstly, we divided the whole 205 accessions into two subgroups: the one that have 8-bp deletion in exon 7 (*badh2*.*1*), and without 8-bp deletion (*Wild*). Then we investigated the extended haplotype homozygosity of *BADH1* and *BADH2* regions to find the evidence for artificial selections. We calculated the EHH for *BAHD2* gene by comparing the haplotypes of fragrant (*badh2*.*1*) and wild type (with functional *BADH2* gene) group. The *badh2*.*1* had a large LD block that extended around the mutation site, while LD decreased rapidly around the wild type alleles (Fig 2B).

**Fig 3:**
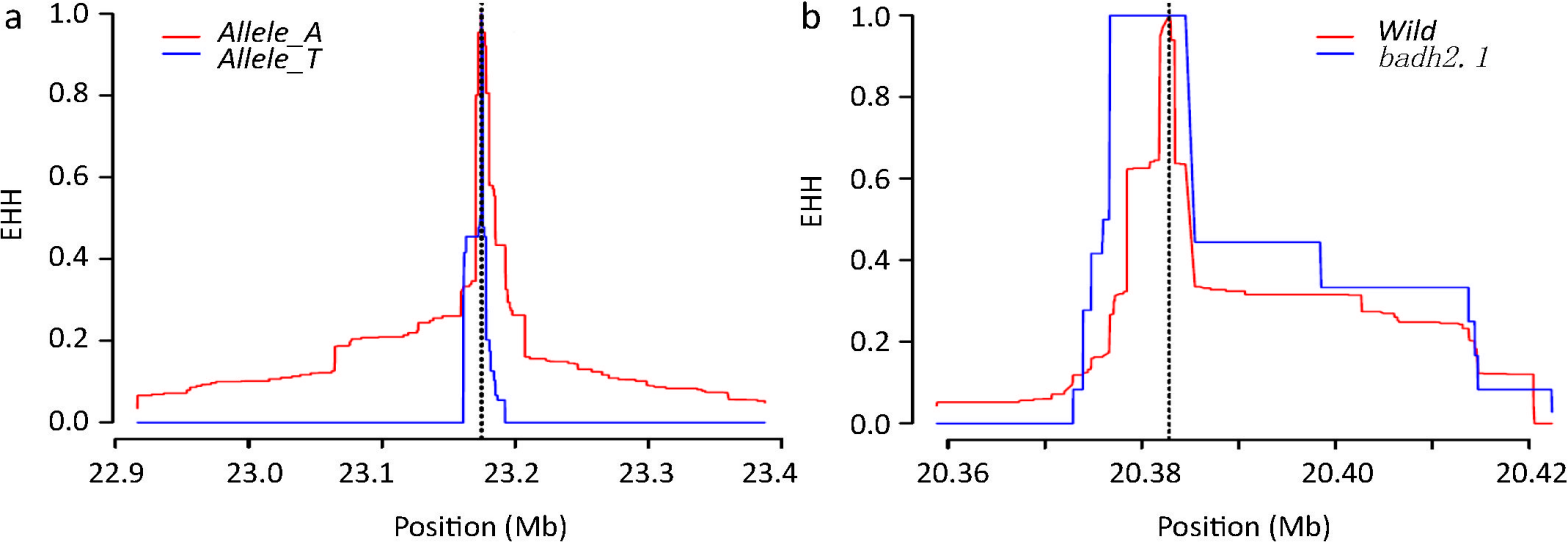
Boxplot of STI (salt tolerance index) for different allele type at P1_1483_ (T/A).

Since the P1_1483_ (T/A) allele was significantly correlated with salt tolerance, we separated the 205 individuals into two subgroups, one included “T” allele (*Allele_T* group), another one included “A” allele (*Allele_A* group). STI values of the “T” allele subgroup were significantly higher than the “A” allele subgroup (P<0.05; Fig 3). The EHH study for the *BADH1* gene was compared the extended haplotypes of (P1_1483_ A) and wide type (P1_1483_ T). However, these two groups carrying A or T alleles did not exhibit large difference, both of them did not show large extended LD block, reducing rapidly around P1_1483_ (Fig 2A).

### LD block, nucleotide diversity and Tajima’s D analysis in different subpopulations

**Fig 4:**
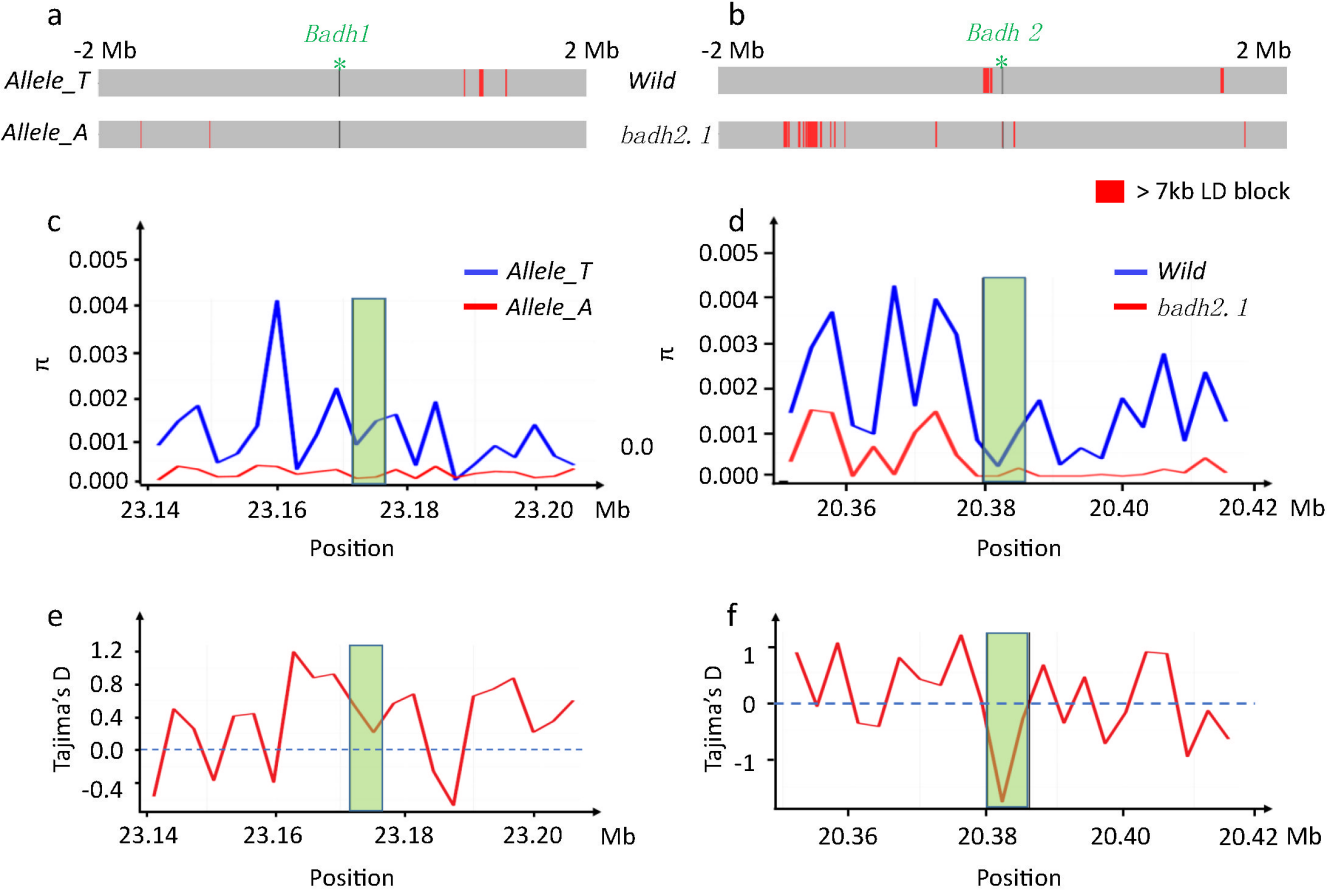
LD block, nucleotide diversity and Tajima’s D in *BADHs* regions (Green part are the gene regions). (a) LD block of *BADH1* at different groups. (b) LD block of *BADH2* at different groups (badh2.1 has 8bp deletion in exon 7). (c) Nucleotide diversity of *BADH1* region with 3kb slide window. (d) Nucleotide diversity of *BADH2* region with 3kb slide window. (e) Tajima’s D of *BADH1* region with 3kb slide window. (f) Tajima’s D of *BADH2* region with 3kb slide window.

In order to find more evidence whether the domestication rate for *BADH1* and *BADH2* are different, we analyzed the LD block, nucleotide diversity and Tajima’s D for each subgroup. For *BADH2*, it clearly showed that wild group has no block in *badh2* gene region, while *badh2*.*1* group had the block which over 7 kb in the gene region (Fig 4B). The nucleotide diversity study showed that wild group had higher diversity than *badh2*.*1* in both the flanking 2 Mb of *BADH2* and *BADH2* gene region. Interestingly, the nucleotide diversity in *BADH2* gene region was reduced in two subgroups. The wild type was still higher than *badh2*.*1* and the diversity of *badh2*.*1* was almost as low as 0 (Fig 4D). Using 205 individual’s sequence, we calculated the Tajima’s D value in *BADHs* regions. It is clearly showed that in *BADH2* region the Tajima’s D value (less than -1) was significantly biased from the balance (Fig 4F). These three results strongly suggested that *badh2*.*1* had been artificially selected during the rice domestication.

On the other hand, we failed to find the large LD block in *BADH1* region in both *Allele_T* group and *Allele_A* group (Fig 4A). The nucleotide diversity study showed that the *Allele_T* group had higher diversity than *Allele_A* in both flanking region and gene region of *BADH1* (Fig 4C), but *Allele_A* group had low diversity level in this region. The Tajima’s D value also kept a stationary value in the *BADH1* gene region (Fig 4E). From these three analyses, we are failed to get any clear evidence that *BADH1* was also selected by human beings, suggesting that *BADH1* is not a domesticated gene in rice.

## Discussion

### Polymorphism and association study

*BADH2* was widely known as the gene associated with fragrance in rice [11]. As the homologous gene of *BADH2*, *BADH1* is considered to be the candidate gene controlling fragrance or salt tolerance in rice. Recently, Fitzgerald et al. (2010) suggested that *BADH2* is also responsible for salt stress tolerance. The relationship between these two *BADH* genes and salt stress tolerance or fragrance in rice is unclear. Various studies have reported conflicting results [1,2,5]. These inconsistencies could be attributable to the differences either in rice germplasm materials or growth stages that they investigated for their studies.

Here, we used whole genome resequencing data to study *BADH1* and *BADH2* genes. For *BADH1*, we found a total of 10 genetic variants, seven SNPs were in the exon region and two SNPs and one insertion were in the untranslated region (S1 Table). Among the seven SNPs in exon region, four of them were novel (S1 Fig). It was observed that four SNPs and three insertions were detected in the UTR of *BADH2*. Three SNPs, two deletions and one insertion were located in the exon region of *BADH2*, extending the number of *BADH2* exonic alleles from 15 to 18 (S2 Table and S2 Fig) [8–11,13].

After removing the accessions with missing sequence information, we finally discovered eight alleles in *BADH1*, and eleven alleles in *BADH2* from the 205 accessions (S3 Table). Eight alleles in *BADH1* generated 7 haplotypes and eleven alleles result in 9 haplotypes in *BADH2* region (S3 Table). We performed the GLM based association study for STI using19 alleles. As a result, we discovered that P1_1483_ (T/A), P1_3605_ (C/A) in translated region and P1_4811_ in the 3’UTR were highly correlated with STI with, *P* < 10^−4^ and *R* ^*2*^ around 0.12 (Table 1 and Fig 1). However, similar correlations were not found for *BADH2* (Table 1), indicating *BADH2* may not be associated with salt tolerance.

Fitzgerald et al. (2008) reported that the expression level of *BADH1* in leaf tissue is highly associated in response to salt treatment at the seedling stage. In contrast, such relationship between *BADH2* transcript levels and salt treatment has not been found yet. More recently, however, Fitzgerald et al. (2010) did show a difference between fragrant (*badh2*) and non-fragrant (*BADH2*) rice for the ability to produce mature seed in the presence of salt. Tang et al. (2014) proved that *BADH1* was associated with rice salt (NaCl) tolerance at seedling stage by using RNAi-directed knock down of *BADH1* [32]. Thus, *BADH1* and *BADH2* are both suggested to be responsible for salt stress tolerance in rice, but, possibly, at different growth stages. Since our STI was tested at germination stage, we extended the conclusion that *BADH1* is response for rice salt stress at germination and seedling stage, while *BADH2* may be accountable at the reproductive stage.

P2_3036_ (8 bp deletions) and P2_5390_ (C/T) were the main alleles that explained the aroma variation with 74% and 23% respectively (S4 Table), which are consistent with previous studies in that the lack of function of *BADH2* has a significant effect on rice aroma [8,11]. However, we found no association between *BADH1* and aroma (S4 Table).

Singh et al. (2010) reported that there’s no association between salt tolerance and common *BADH1* but few exceptions. None the less, our results are rather consistent with the result from Fitzgerald et al. (2008), demonstrating that *BADH1* (and not *BADH2*) contributes to salt stress tolerance. However, we cannot exclude the possibility that these contradictions may be resulted from the different conditions undertaken.

### Domestication of *BADH1* and *BADH2*

Domestication of crops has historically been critical for human reproduction and development. The domestication of rice were explained by several models [11]. The recent increase in molecular marker density through next generation sequencing (NGS) technology has facilitated to build more solid hypotheses on the domestication process of rice [11,33]. Investigating the domestication history of a specific gene is one way to decipher the history of rice and its impact on humans [11]. An EHH study of *BADH2* led by Kovach et al. (2009) suggested that *BADH2* is one of the domesticated genes responsible for rice fragrance. In this study, we attempted to determine if *BADH1* is also a domesticated gene.

The association mapping study identified three alleles (P1_1483_, P1_3605_ and P1_4811_) in *BADH1* highly correlated with STI. These three alleles were consistently present together in the rice genome. We selected P1_1483_ (T/A) as the key allele for an evolution study of *BADH1*. To validate our method, we tested the domestication signal of *BADH2* using the same method (EHH) as Kovach et al. (2009). Our EHH test result was similar to the results of Kovach et al. (2009). The fragrant accessions which carrying 8 bp deletions in exon 7 showed a large LD block extended around the mutation site, while the wild type alleles relatively reduced rapidly (Fig 2B). To obtain more clear evidence, we further investigated LD block, nucleotide diversity, and Tajima’s D for the 4-Mb flanking regions of *BADHs*, which had always been tested for mutual verification for a domestication process [11,18,19,34]. For *BADH2*, LD block study showed that there were a lot of LD blocks in the fragrant rice group (*badh2*.*1*) over 7 kb, while fewer LD blocks were found in wild type group (Fig 4B). The diversity study suggested that all groups show reduced diversity in *BADH2* gene region, but the *badh2*.*1* approaches zero for this region (Fig 4D). Furthermore, the Tajima’s D test showed a significantly deviated signal in *BADH2* region (Fig 4F). All of those results support the conclusions that *badh2*.*1* was domesticated, which is totally consistent with previous study [11].

Employing the same tests, finding clear domesticated signals for *BADH1* was difficult. We failed in finding LD blocks in *BADH1* for the “*Allele_A*” and “*Allele_T*” groups (Fig 4A). The diversity of *Allele_A* group was consistently low without any violent swings in *BADH1* gene region (Fig 4C). Moreover, Tajima’s D value was not significant for *BADH1* (Fig 4E). Lastly, EHH was significantly diminished in the two *BADH1* groups (Fig 2A). Thus, we concluded that *BADH1* had not been domesticated in Asian rice.

Interestingly, Wang et al. (2014) found that the genetic diversity is strongly reduced in the *BADH1* region of *O*. *glaberrima*, indicating *BADH1* was possibly domesticated in *O*. *glaberrima* [35]. *O*. *glaberrima* is widely known to be better at tolerating biotic or abiotic stress than *O*. *sativa* [36]. As a gene associated with abiotic stress tolerance, *BADH1* was likely domesticated in *O*. *glaberrima*, which is not the case for *O*. *sativa* [36]. Our results supports that the *BADH1* is responsible for salt stress in rice germination stage, explaining 12% variation of phenotype for salt stress in *O*. *sativa* (Table 1). However, this effect might not be strong enough to be selected during the evolution of Asian cultivated rice.

## Supporting Information

S1 Fig *BADH1* allelic diversity in exon region. *badh1*.*3*, *badh1*.*5* and *badh1*.*6* were detected by previous study [1]. *badh1*.*1*, *badh1*.*2*, *badh1*.*4* and *badh1*.*7* were detected in this study.
(DOCX)

S2 Fig *BADH2* allelic diversity. *badh2*.*1~badh2*.*2* [11]; *badh2*.*11~badh2*.*12*, *badh2*.*14* [10]; *badh2*.*13* [16]; *badh2*.*15* [12]. *badh2*.*16~badh2*.*18* were detected in this study.
(DOCX)

S1 Table Transcribed polymorphism in *BADH1*. (DOCX)

S2 Table Transcribed polymorphism in *BADH2*. (DOCX)

S3 Table Haplotype of *BADH1* and *BADH2*, and phenotype. (Excel)
(XLSX)

S4 Table The association study of aroma and candidate regions. (DOCX)
