# Supplementary material for: Resequencing Reveals Different Domestication Rate for BADH1 and BADH2 in Rice (Oryza sativa)
Source: PLoS One. 2015 Aug 10;10(8):e0134801. doi: 10.1371/journal.pone.0134801 (PMC4530958; doi:10.1371/journal.pone.0134801)
Supplement: S1 Fig — badh1.3, badh1.5 and badh1.6 were detected by previous study [1]. badh1.1, badh1.2, badh1.4 and badh1.7 were detected in this study. (DOCX) [file pone.0134801.s001.docx]

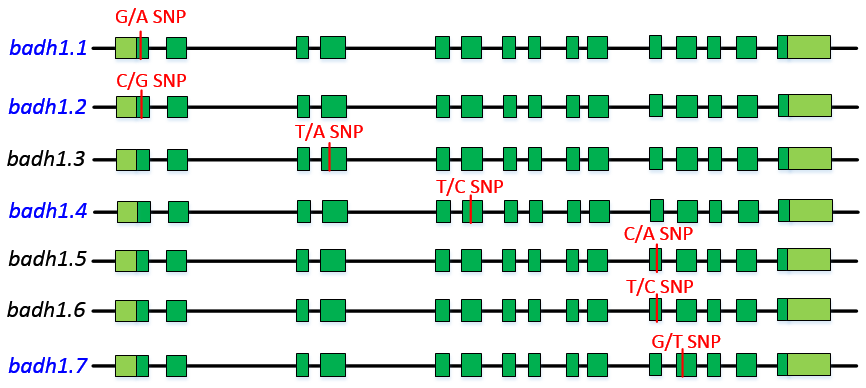


**S1 Fig. *BADH1* allelic diversity in exon region.** *badh1.3, badh1.5* and *badh1.6* were detected by previous study [1]. *badh1.1*, *badh1.2*, *badh1.4* and *badh1.7* were detected in this study.
