# Supplementary material for: Resequencing Reveals Different Domestication Rate for BADH1 and BADH2 in Rice (Oryza sativa)
Source: PLoS One. 2015 Aug 10;10(8):e0134801. doi: 10.1371/journal.pone.0134801 (PMC4530958; doi:10.1371/journal.pone.0134801)
Supplement: S2 Fig — badh2.1~badh2.2 [11]; badh2.11~badh2.12, badh2.14 [10]; badh2.13 [16]; badh2.15 [12]. badh2.16~badh2.18 were detected in this study. (DOCX) [file pone.0134801.s002.docx]

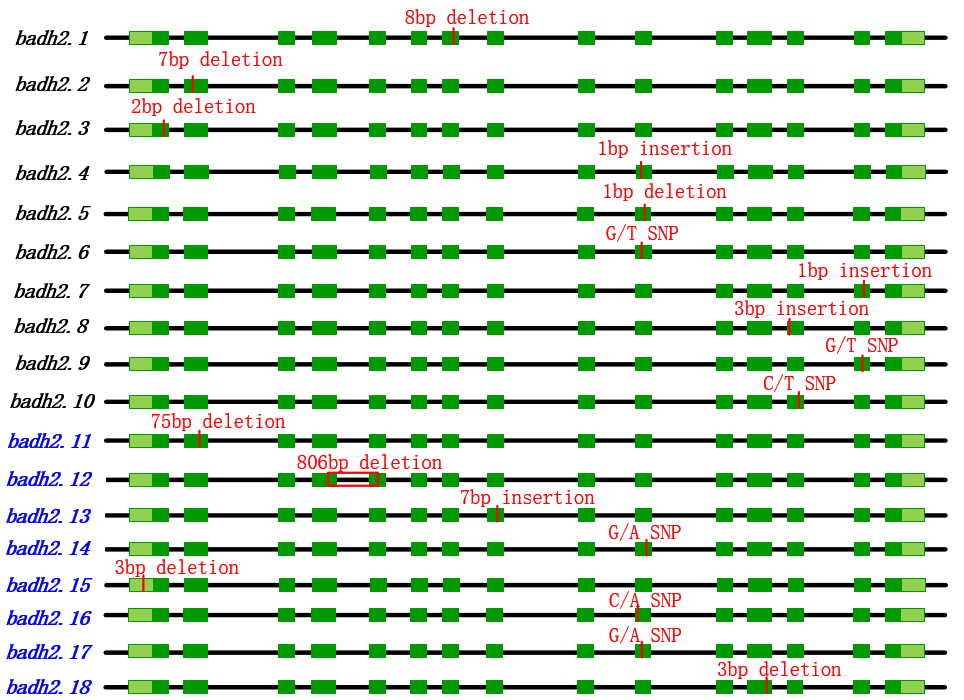


**S2 Fig. *BADH2* allelic diversity.** *badh2.1~badh2.2* [11]; *badh2.11~badh2.12*, *badh2.14* [10]; *badh2.13* [36]; *badh2.15* [12]. *badh2.16~badh2.18* were detected in this study.
