# Supplementary material for: Resequencing Reveals Different Domestication Rate for BADH1 and BADH2 in Rice (Oryza sativa)
Source: PLoS One. 2015 Aug 10;10(8):e0134801. doi: 10.1371/journal.pone.0134801 (PMC4530958; doi:10.1371/journal.pone.0134801)
Supplement: S1 Table — (DOCX) [file pone.0134801.s003.docx]

S1 Table. Transcribed polymorphism in *BADH1*

| Position | Alleles | Amino acid | CAN | WAN |  |
| --- | --- | --- | --- | --- | --- |
| 11 | A/T |  | 0 | 1(H) | 5'UTR |
| 46 | C/G |  | 19 | 0 | 5'UTR |
| 87 | 6 bp insertion |  | 4 | 0 | 5'UTR |
| 101 | C/A |  | 1 | 0 | 5'UTR |
| 141 | G/A | R/H | 4 | 0 | Exon 1 |
| 181 | C/G | L/L | 14+2(H) | 0 | Exon 1 |
| 378 | C/T | L/L | 0 | 1 | Exon 2 |
| 438 | G/C | A/A | 0 | 1 | Exon 2 |
| 1232 | T/A | D/E | 0 | 1(H) | Exon 3 |
| 1244 | T/C | Y/Y | 0 | 1(H) | Exon 3 |
| 1483 | T/A | N/K | 47 | 8 | Exon 4 |
| 2415 | T/C | T/T | 1 | 0 | Exon 6 |
| 3063 | C/T | N/N | 0 | 1(H) | Exon 9 |
| 3193 | A/G | R/R | 0 | 1 | Exon 10 |
| 3605 | C/A | Q/K | 47 | 8 | Exon 11 |
| 3612 | T/C | I/T | 1 | 0 | Exon 11 |
| 3625 | C/T | I/I | 0 | 2(H) | Exon 11 |
| 3672 | G/T | R/L | 0 | 1(H) | Exon 11 |
| 3796 | T/C | T/T | 0 | 1(H) | Exon 12 |
| 3883 | G/T | E/D | 2 | 1+1(H) | Exon 12 |
| 4568 | A/G |  | 0 | 1(H) | 3'UTR |
| 4695 | T/C |  | 0 | 1(H) | 3'UTR |
| 4811 | C/A |  | 47 | 8 | 3'UTR |

CAN: Cultivated rice accession numbers (from 295 cultivated rice accessions).

WAN: Wild rice accession numbers (five *O. rufipogon* and five *O. nivara*).

H：Heterozygous.
