# Supplementary material for: Resequencing Reveals Different Domestication Rate for BADH1 and BADH2 in Rice (Oryza sativa)
Source: PLoS One. 2015 Aug 10;10(8):e0134801. doi: 10.1371/journal.pone.0134801 (PMC4530958; doi:10.1371/journal.pone.0134801)
Supplement: S2 Table — (DOCX) [file pone.0134801.s004.docx]

S2 Table. Transcribed polymorphism in *BADH2*.

| Pos. | Alleles | Amino acid | CAN | WAN | Locus |
| --- | --- | --- | --- | --- | --- |
| 14 | 1bp insertion |  | 5 | 1(H) | 5'UTR |
| 24 | C/T |  | 5 | 0 | 5'UTR |
| 36 | 1bp deletion |  | 52 | 0 | 5'UTR |
| 41 | 5bp insertion |  | 6 | 0 | 5'UTR |
| 42 | C/T |  | 6 | 1 | 5'UTR |
| 49 | C/A |  | 57+3(H) | 3+1(H) | 5'UTR |
| 115 | G/T |  | 6 | 1 | 5'UTR |
| 205 | C/G | A/G | 0 | 2(H) | Exon 1 |
| 2658 | C/T | A/V | 0 | 1(H) | Exon 5 |
| 2853 | A/C | Leu/Leu | 0 | 1(H) | Exon 6 |
| 3036 | 8bp deletion |  | 9 | 0 | Exon 7 |
| 4488 | C/A | A/E | 6 | 0 | Exon 10 |
| 4528 | G/A | Val/Val | 1+1(H) | 0 | Exon 10 |
| 5171 | C/T | Val/Val | 0 | 1(H) | Exon 12 |
| 5240 | 3bp deletion | E deletion | 1 | 0 | Exon 12 |
| 5390 | C/T | A/V | 3 | 0 | Exon 13 |
| 5772 | 1bp insertion |  | 1 | 0 | Exon 14 |
| 6038 | C/T |  | 0 | 1(H) | 3’UTR |

CAN: Cultivated rice accession numbers (from 295 cultivated rice accessions).

WAN: Wild rice accession numbers (five *O. rufipogon* and five *O. nivara*).

H：Heterozygous.
