# Supplementary material for: Resequencing Reveals Different Domestication Rate for BADH1 and BADH2 in Rice (Oryza sativa)
Source: PLoS One. 2015 Aug 10;10(8):e0134801. doi: 10.1371/journal.pone.0134801 (PMC4530958; doi:10.1371/journal.pone.0134801)
Supplement: S4 Table — (DOCX) [file pone.0134801.s006.docx]

S4 Table. The association study of aroma and candidate regions.

| Marker | Locus | Locus_pos | marker_F | marker_p | Marker R^2^ |
| --- | --- | --- | --- | --- | --- |
| rs-01 | *BADH1* | 46 | 0.39197 | 0.67624 | 0.00387 |
| rs-02 | *BADH1* | 87 | 2.72325 | 0.10044 | 0.01324 |
| rs-03 | *BADH1* | 101 | 0.06189 | 0.80378 | 3.05E-04 |
| rs-04 | *BADH1* | 181 | 0.39197 | 0.67624 | 0.00387 |
| rs-05 | *BADH1* | 1483 | 0.06191 | 0.93999 | 6.13E-04 |
| rs-06 | *BADH1* | 3605 | 0.06191 | 0.93999 | 6.13E-04 |
| rs-07 | *BADH1* | 3883 | 0.06189 | 0.80378 | 3.05E-04 |
| rs-08 | *BADH1* | 4811 | 0.06191 | 0.93999 | 6.13E-04 |
| rs-09 | *BADH2* | 14 | 0.18763 | 0.66536 | 9.23E-04 |
| rs-10 | *BADH2* | 24 | 0.18763 | 0.66536 | 9.23E-04 |
| rs-11 | *BADH2* | 36 | 2.72475 | 0.10035 | 0.01324 |
| rs-12 | *BADH2* | 41 | 0.18763 | 0.66536 | 9.23E-04 |
| rs-13 | *BADH2* | 42 | 0.12443 | 0.72465 | 6.13E-04 |
| rs-14 | *BADH2* | 49 | 1.44944 | 0.23713 | 0.01415 |
| rs-15 | *BADH2* | 115 | 0.12443 | 0.72465 | 6.13E-04 |
| rs-16 | *BADH2* | 3036 | 573.3512 | 4.82E-61 | 0.73852 |
| rs-17 | *BADH2* | 4488 | 0.18763 | 0.66536 | 9.23E-04 |
| rs-18 | *BADH2* | 4528 | 0.06189 | 0.80378 | 3.05E-04 |
| rs-19 | *BADH2* | 5390 | 63.70569 | 1.05E-13 | 0.23886 |

Locus_pos: Physical distance from the transcription initiation site of the gene.
